# Metabolic reprogramming of osteoclasts represents a therapeutic target during the treatment of osteoporosis

**DOI:** 10.1038/s41598-020-77892-4

**Published:** 2020-12-03

**Authors:** Jule Taubmann, Brenda Krishnacoumar, Christina Böhm, Maria Faas, Dorothea I. H. Müller, Susanne Adam, Cornelia Stoll, Martin Böttcher, Dimitrios Mougiakakos, Uwe Sonnewald, Jörg Hofmann, Georg Schett, Gerhard Krönke, Carina Scholtysek

**Affiliations:** 1 Department of Internal Medicine 3, Friedrich Alexander University of Erlangen-Nuremberg (FAU) and Universitätsklinikum Erlangen, Erlangen, Germany; 2 Deutsches Zentrum für Immuntherapie (DZI), University of Erlangen-Nuremberg and Universitätsklinikum Erlangen, Erlangen, Germany; 3 Department of Internal Medicine 5, University of Erlangen-Nuremberg and Universitätsklinikum Erlangen, Erlangen, Germany; 4 Division of Biochemistry, Department of Biology, University of Erlangen-Nuremberg, Erlangen, Germany

**Keywords:** Cell biology, Immunology, Rheumatology

## Abstract

Osteoclasts are specialised bone resorbing cells that control both physiological and pathological bone turnover. Functional changes in the differentiation and activity of osteoclasts are accompanied by active metabolic reprogramming. However, the biological significance and the in vivo relevance of these events has remained unclear. Here we show that bone resorption of differentiated osteoclasts heavily relies on increased aerobic glycolysis and glycolysis-derived lactate production. While pharmacological inhibition of glycolysis did not affect osteoclast differentiation or viability, it efficiently blocked bone resorption in vitro and in vivo and consequently ameliorated ovariectomy-induced bone loss. Our experiments thus highlight the therapeutic potential of interfering with osteoclast-intrinsic metabolic pathways as possible strategy for the treatment of diseases characterized by accelerated bone loss.

## Introduction

Bone is a dynamic tissue undergoing constant remodeling that is orchestrated by osteoclast-mediated bone resorption and osteoblast-mediated bone formation. Osteoclasts (OCs) are formed from precursor cells (OCPs) of the monocyte/macrophage lineage in the presence of macrophage colony-stimulating factor (M-CSF) and receptor activator of NFκB ligand (RANKL)^1,2^. OCs are the primary bone-resorbing cells in both physiological and pathological states and thus play a key role in regulating bone mass. Pathologically enhanced osteoclast activity and accelerated bone resorption are observed during postmenopausal osteoporosis and diseases such as rheumatoid arthritis leading to decreased bone mass and increased risk of fractures^3^.

The molecular events underlying OC differentiation have been extensively studied. RANKL has been identified as key cytokine during osteoclastogenesis and its therapeutic targeting reduces osteoclast numbers and bone resorption during the treatment of osteoporosis. Mechanisms that control and adjust the function of mature osteoclasts are less well understood. However, insights into these pathways would be highly relevant to design novel therapeutic approaches to fine tune osteoclast function.

Recent data indicate that active metabolic reprogramming occurs during RANKL-induced osteoclastogenesis, where these multinucleated cells show an increase in mitochondrial content, biomass and mitochondrial respiration as well as accelerated glycolytic metabolism^4–6^. More recently, human osteoclasts were described to directly increase glycolysis during bone resorption in vitro. However, the in vivo consequences of these events remain unclear^7^. In this study, we used cell culture medium containing bone powder to induce a bone-resorbing phenotype in OCs and to study the consecutive functional and metabolic changes. We observed an active metabolic switch in bone resorbing OCs, characterized by increased glycolysis and lactate production. Notably, both glycolysis and its metabolite lactate were essential for efficient bone resorption. Functional inhibition of glycolysis by 2-Desoxy-D-glucose (2-DG) blocked bone resorption by OCs, which could be rescued by supplementation of either pyruvate or lactate suggesting lactate as an active metabolic intermediate that drives bone resorption. In accordance with a key role of glycolysis during OC-mediated bone resorption in vivo, we observed effective amelioration of ovariectomy-induced bone loss in mice that received 2-DG or a small molecular inhibitor of lactate dehydrogenase A (LDH-A).

## Results

### Activation of osteoclasts is paralleled by increased aerobic glycolysis

**Figure 1:**
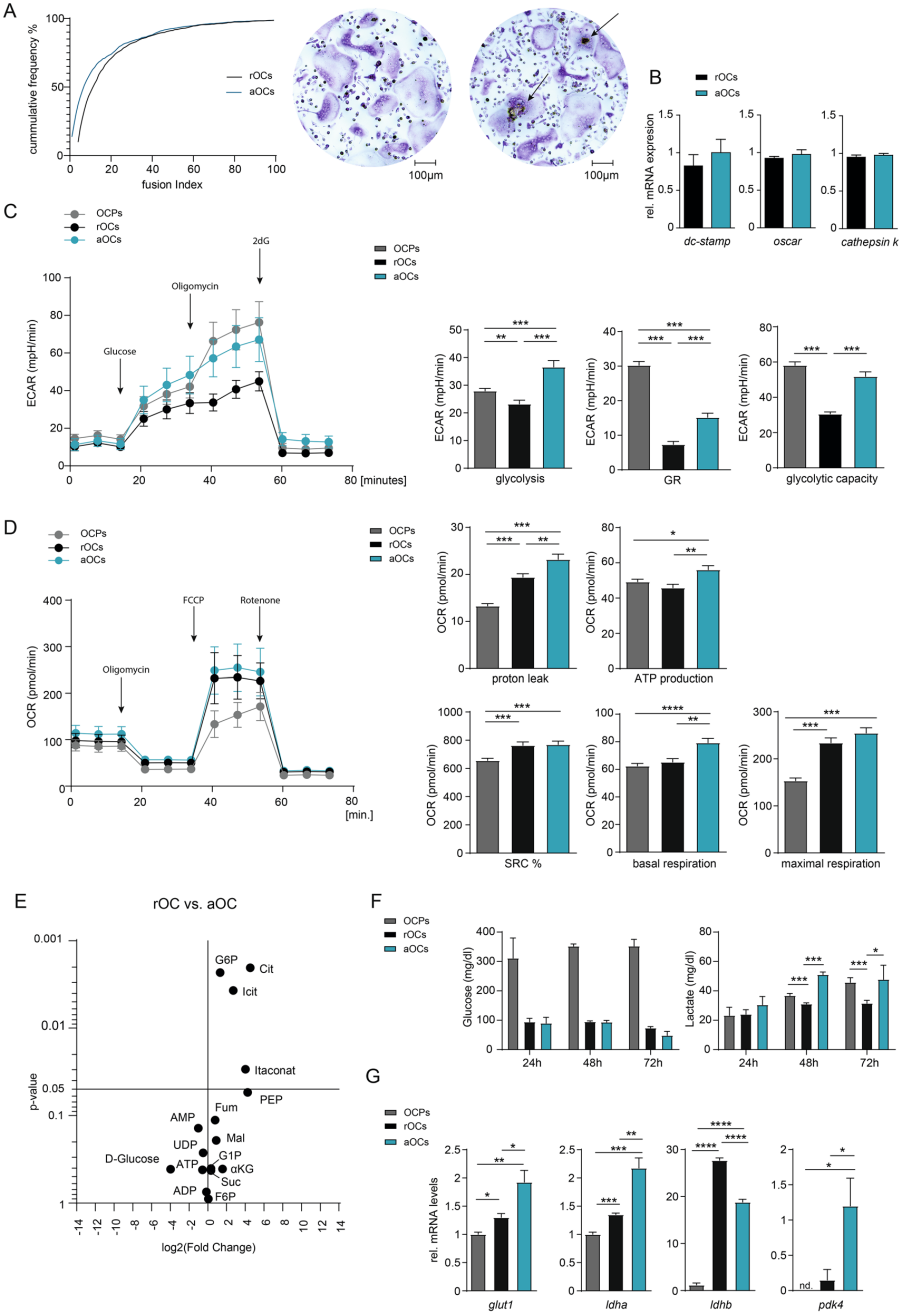
Activation of osteoclasts is paralleled by increased glycolysis. (**A**) Fusion index (left panel) and representative TRAP staining (right panel) of OCPs, rOCs and aOCs on day 4 of osteoclast cell culture. (**B**) Quantitative RT-PCR measuring relative mRNA levels of *dc-stamp*, *oscar* and *cathepsin k*, normalized on beta-actin after day 3 of osteoclast cell culture in rOCs and aOCs. (**C**) Extracellular acidification rate (ECAR) including glycolysis, glycolytic capacity and glycolytic reserve (GR), measured with an extracellular flux (XF) analyzer in rOCs and aOCs after 48 h. (**D**) Oxygen consumption rate (OCR) including basal and maximal respiration rate, ATP production, proton leak and spare respiratory capacity (SRC), measured with an extracellular flux (XF) analyzer in OCPs, rOCs and aOCs after 48 h. (**E**) Mass spectrometry-based analysis of the abundance of indicated metabolites in rOCs and aOCs (**F**) Glucose consumption and lactate accumulation in culture media at indicated time points of OC cell culture comparing OCPs, rOCs and aOCs. (**G**) Quantitative RT-PCR determining relative mRNA levels of glucose transporter 1 (*glut1*), Lactate dehydrogenase A (*ldha*), Lactate dehydrogenase B (*ldhb*) and Pyruvate dehydrogenase lipoamide kinase isozyme 4 (*pdk4*), normalized on beta-actin after indicated time points of OC cell culture.

To analyse changes in the metabolic activity of bone resorbing OCs, we isolated murine bone marrow-derived monocytes as OCPs and cultured these cells in M-CSF and RANKL containing medium in the absence and presence of bone powder to simulate a bone resorbing metabolic state in such “activated” OCs (aOCs). The application of bone powder medium did not affect OC fusion capacity, OC number or the expression of OC differentiation marker genes, such as *dc-stamp*, *oscar* and *cathepsin k* (Fig. 1A,B). By performing extracellular flux assays, we compared changes in the extracellular acidification rate (ECAR) as measure of the glycolytic activity between OPCs, non-activated “resting” OCs (rOCs) and bone resorbing aOCs. OC differentiation resulted in a reduced glycolytic activity in rOCs in comparison to OCPs (Fig. 1C). However, aOCs displayed increased glycolysis, glycolytic reserve (GR) and glycolytic capacity when compared to rOCs, confirming that bone resorption was paralleled by accelerated extracellular acidification and an enhanced aerobic glycolysis (Fig. 1C). Both rOCs and aOCs populations revealed marked elevation of their oxygen consumption rate (OCR), indicated by increased spare respiratory capacity (SRC) and maximal respiration. Moreover, rOCs and aOCs showed an elevated proton leak and basal respiration demonstrating increased mitochondrial respiration during OC differentiation and bone resorption (Fig. 1D).

In addition, we performed metabolic profiling of rOCs and aOCs. In accordance with the data derived from the extracellular flux assays, we observed an increase of metabolites of both glycolysis and the Krebs cycle such as glucose 6-phosphate (G6P) or citrate (Cit), respectively, upon initiation of bone resorption in aOCs (Fig. 1E). Direct measurement of glucose consumption and lactate accumulation in the growth media of rOCs and aOCs revealed that both OC populations substantially consumed glucose. However, lactate production was only significantly elevated in bone-resorbing aOCs, which was in accordance with their enhanced state of aerobic glycolysis (Fig. 1F). When we analysed the metabolic gene expression profile, we found several genes of the glycolytic pathway induced in aOCs (Fig. 1G). In particular, expression of *glut1*, *ldha* and *pdk4* were increased and provided an explanation for the increase in the rate of glycolysis and enhanced lactate production. On the contrary, *ldhb*, which catalyses the conversion of lactate and NAD + to pyruvate, was downregulated in aOCs, indicating a metabolic shift towards active aerobic glycolysis and lactate production during bone resorption.

### Glycolysis-derived lactate essentially supports bone resorption

**Figure 2:**
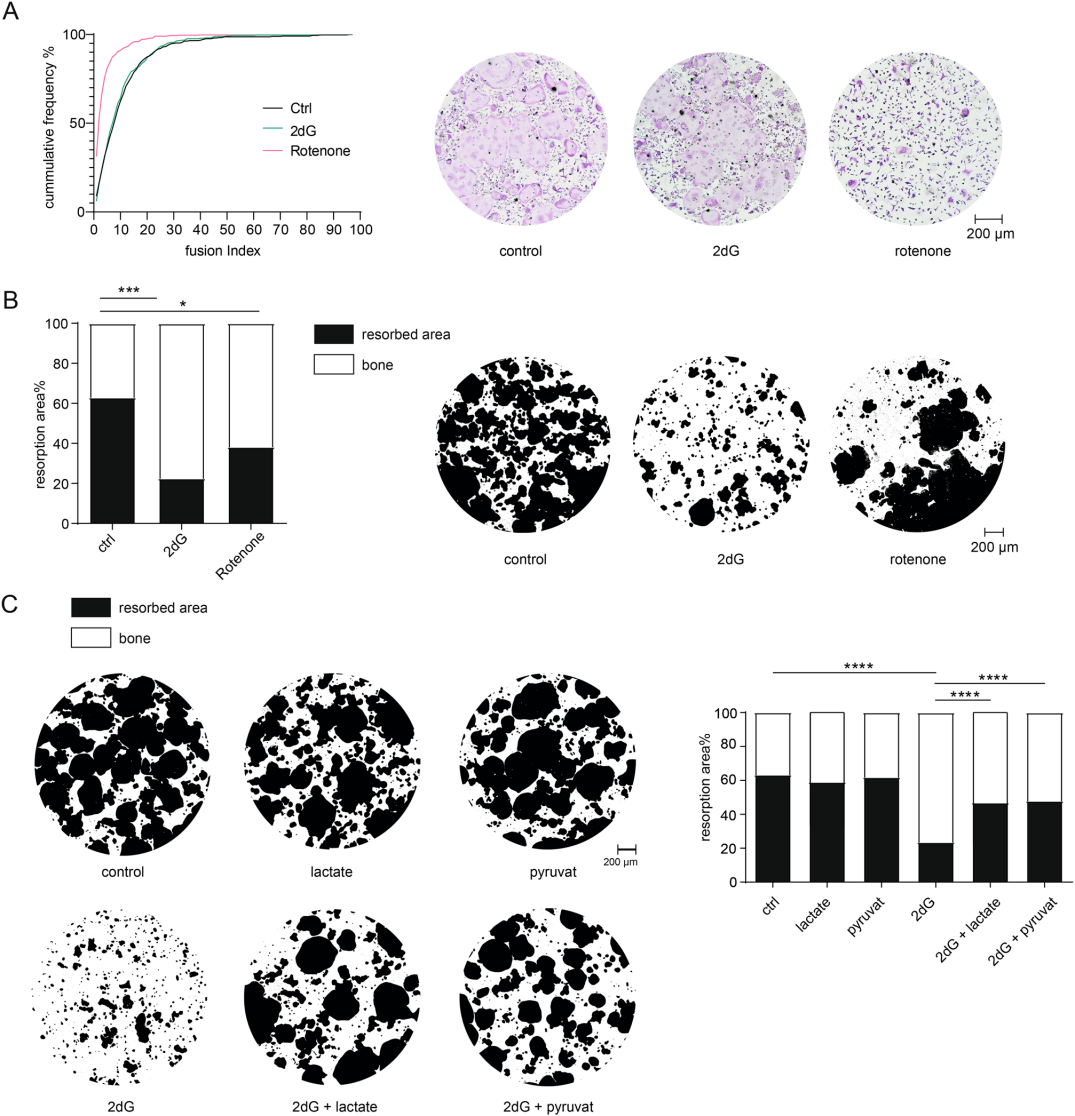
Glycolysis routes bone resorptive activity in vitro. (**A**) Fusion index (left) and representative TRAP staining of OCs differentiated in the absence or presence of the glycolysis inhibitor 2-Deoxy-d-glucose (2dG; 10 µM) or rotenone (0.02 µM) on day 3 of OC culture. (**B**,**C**) OCs were generated on bone resorption plates for 5 days and stimulated with 2dG (10 µM) or rotenone (0.02 µM) in the presence or absence of lactate (5 mM) or pyruvate (2 mM) as indicated. The percentage of the resorbed area was quantified via Photoshop and pictures show representative images of resorption pit formation.

Next, we aimed to address the functional consequences of the observed metabolic reprogramming of aOCs including the increase in aerobic glycolysis and lactate production on osteoclast differentiation and bone resorption. We therefore isolated monocytes, assessed OC differentiation and quantified their bone resorbing capacity in the presence of specific inhibitors of oxidative phosphorylation and glycolysis, respectively. Notably, 2dG-mediated inhibition of glycolysis efficiently blocked bone resorption, but did not affect OC differentiation and fusion, whereas rotenone as an inhibitor of mitochondrial complex I, decreased osteoclastogenesis but did only moderately affect resorption activity (Fig. 2A,B). These results indicated that aerobic glycolysis in OCs was essential for OC activation and bone resorption, but did not affect earlier steps of osteoclastogenesis such as differentiation and fusion events. To determine downstream mediators and metabolites that were responsible for the glycolysis-mediated increase in bone resorption, we assessed the impact of the glycolysis products lactate and pyruvate on the bone resorption capacity of aOCs in the absence and presence of 2dG. While lactate and pyruvate alone did not affect the bone resorbing activity of aOCs, both metabolites rescued the decreased bone resorption caused by inhibition of glycolysis via 2dG (Fig. 2C), which suggested lactate as the active metabolite mediating bone resorption in response to enhanced glycolysis in aOCs.

### Inhibition of glycolysis and lactate production ameliorate ovariectomy-induced bone loss

**Figure 3:**
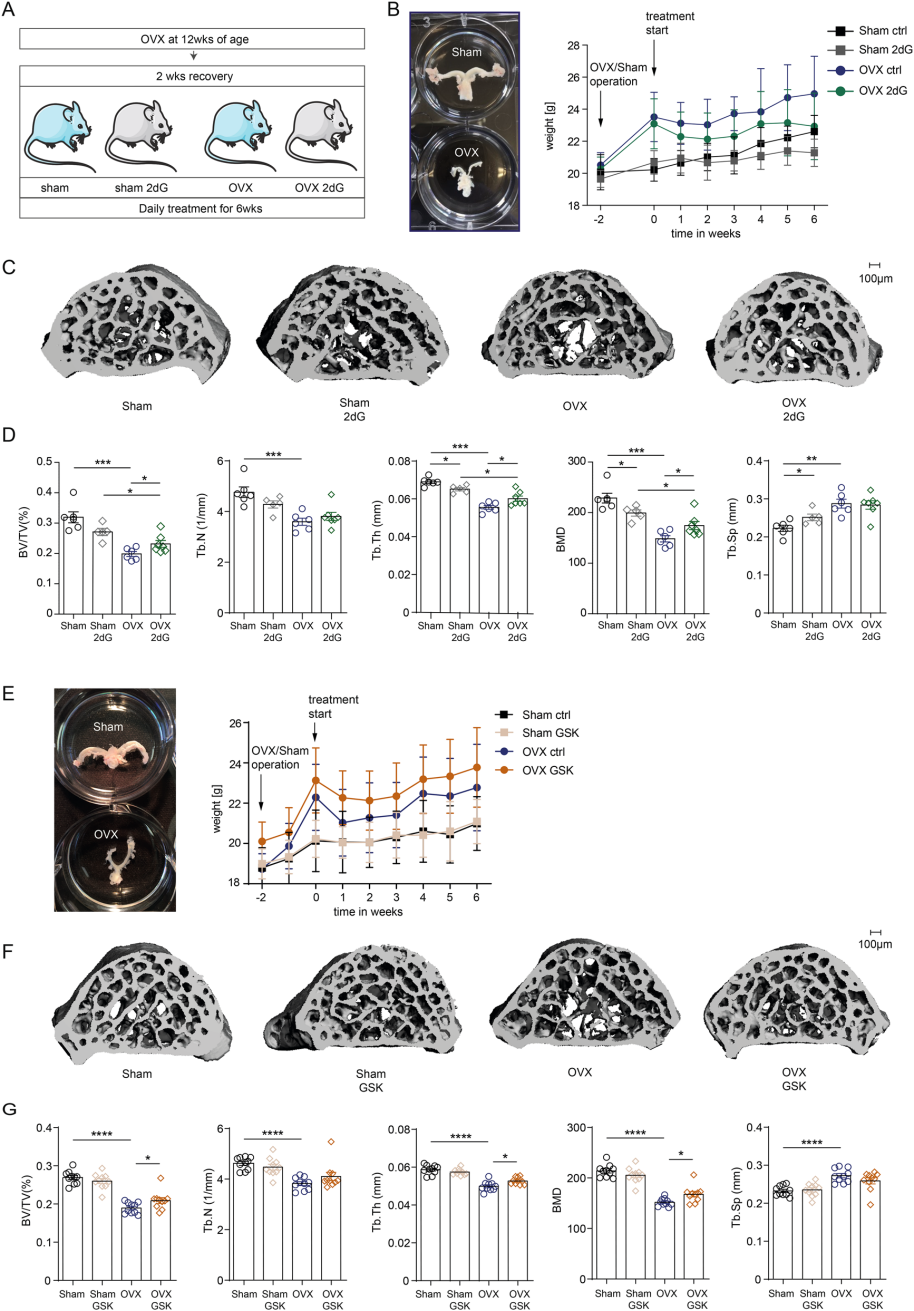
Inhibition of glycolysis and lactate production ameliorates OVX-induced bone loss. (**A**) Schematic diagram illustrating the postmenopausal mouse model of ovariectomy-induced bone loss and treatment strategies. (**B**) Weight course in ovariectomized (OVX) or sham-operated mice after treatment with 2dG treatment or treatment with a vehicle (aqua injectabile), respectively. (**C**) Representative images show spinal architecture by μCT reconstruction. (**D**) µCT measurements of indicated parameters of spinal microarchitecture including bone volume to total volume (BV/TV), trabecular number (Tb.N.), trabecular thickness (Tb.Th.) and trabecular separation (Tb.Sp.) in ovariectomized (OVX) or sham-operated mice after treatment with 2dG, respectively. (**E**) Demonstration of adnexe from Sham mouse and ovariectomized mouse 8 weeks after surgery and course of weight gain in ovariectomized (OVX) or sham-operated mice after treatment with the LDHA inhibitor GSK2837808A (GSK) or a vehicle (DMSO). (**F**) Representative images show spinal architecture of indicated groups by μCT reconstruction. (**G**) µCT measurements of indicated parameters of spinal microarchitecture including bone volume to total volume (BV/TV), trabecular number (Tb.N). trabecular thickness (Tb.Th.) and trabecular separation (Tb.Sp) in ovariectomized (OVX) or sham-operated mice after treatment with GSK or the vehicle.

To assess the potential of OC-mediated glycolysis as therapeutic target for diseases characterized by a pathologically enhanced bone resorption, we decided to study the effect of blocking glycolysis and lactate production during a mouse model of ovariectomy-induced bone loss. Therefore, we treated mice that displayed an established osteoporosis (treatment start 2 weeks post ovariectomy) with the glycolysis inhibitor 2dG for 6 additional weeks before bone structure was analysed by μCT (Fig. 3A). 2dG treatment was well tolerated. As expected, mice that had undergone ovariectomy displayed an increased weight gain over the study period of 8 weeks, which was slightly decreased by treatment with 2dG (Fig. 3B). Analysis of the trabecular microstructure of the spine showed that 2dG slightly decreased bone density in the sham-operated group, whereas we observed an increase in bone density upon 2dG treatment in mice that had undergone ovariectomy (Fig. 3C,D). 2dG treatment resulted in an increased bone volume/total volume (BV/TV), an increased bone mineral density (BMD) and an increased trabecular thickness in ovariectomized mice, which was in accordance with a block of pathologic bone resorption in these mice.

We additionally validated these results and our in vitro findings in a second round of experiments where we treated mice with the LDHA inhibitor GSK2837808A to selectively interfere with lactate production in vivo. GSK2837808A inhibited the bone resorbing activity of osteoclasts to a similar extend as 2dG without cytotoxic effects in vitro (Suppl. Fig. 1A,B). Treatment was started 2 weeks after ovariectomy in mice that already had developed bone loss (Fig. 3E). In accordance with our previous results, block of lactate synthesis resulted in a slight decrease in bone density in sham- operated mice, whereas GSK2837808A treatment resulted in a significant improvement of ovariectomy-induced bone loss (Fig. 3F,G). Here, we observed an increased BV/TV, an increased trabecular thickness as well as an increased BMD in ovariectomized mice that had received GSK2837808A.

## Discussion

Increasing data highlight the mutual influence of cellular metabolic processes and the functional properties of innate and adaptive immune cells. Among others, macrophages were shown to undergo a defined metabolic reprogramming in response to different activation stimuli. Pro-inflammatory classically-activated macrophages e.g. increase their glycolytic activity and shut down their mitochondrial respiration, whereas anti-inflammatory alternatively-activated macrophages show an increase in mitochondrial respiration. These contrasting metabolic adaptions of individual macrophage subsets seem to support distinct activation and differentiation programs, thereby promoting either onset or resolution of inflammation^8^. Our current data confirm that osteoclast, as mononuclear phagocyte-derived cells that settle the bone microenvironment, undergo specific metabolic adaptions during differentiation and bone resorption as well.

Notably, and in contrast to many other immune cells, bone resorbing and activated osteoclasts are not characterized by an isolated, but by a simultaneous increase of both glycolytic activity and mitochondrial respiration when compared to resting osteoclasts. This observation suggests that the process of bone resorption substantially increases the metabolic and energetic requirements of osteoclasts. This adaptation might be merely linked to an increased demand in energy. Alternatively, it points to the necessity of a provision of specific and essential metabolites that derive from glycolysis and/or mitochondrial respiration and support the functional properties of osteoclasts during bone resorption. Our data primarily support the latter scenario and suggest that glycolysis-derived lactate is essential for the efficient resorption of bone by osteoclasts. Block of oxidative phosphorylation, in turn, only moderately affected bone resorption, although osteoclast differentiation was clearly impaired. Block of glycolysis by 2dG or block of the conversion of pyruvate to lactate by the LDHA-inhibitor GSK2837808A accordingly interfered with regular bone resorption, which could be rescued by the restoration of regular lactate levels. The exact role of lactate during this process remains unclear at the moment. Our in vitro experiments show a substantial increase in extracellular lactate upon initiation of bone resorption. A likely possibility is therefore a role of lactate during the acidification of the osteoclast resorption pit, which is an essential step during bone resorption by osteoclasts, facilitates the activation of acid proteases that brake down bone matrix, and additionally dissolves bone minerals.

Our data additionally show that blocking glycolysis and lactate production represents efficient strategies to interfere with pathological osteoclast-mediated bone loss during ovariectomy as a model of postmenopausal osteoporosis that is usually characterized by increased differentiation and activation of osteoclasts^9^. Although such a strategy significantly increased bone mass in osteoporotic mice, healthy mice displayed a slightly decreased bone density upon block of glycolysis. Although the underlying reasons remain to be elucidated, block of glycolysis likely affects other cell types such as osteoblasts and osteocytes that are equally relevant for a regular bone homeostasis. Especially osteoblast are dependent on glycolysis to form new bone^10^, although they equally require mitochondrial respiration and fatty acids to mineralize newly formed extracellular matrix^11^.

Taken together, our data identify aerobic glycolysis and osteoclast-mediated lactate production as essential steps during bone resorption and suggest that blocking glycolysis and lactate production in osteoclasts represents a potential therapeutic strategy during diseases that are characterised by accelerated osteoclast-mediated bone loss such as postmenopausal osteoporosis and rheumatoid arthritis.

## Methods

The authors confirm that all methods were carried out in accordance with relevant guidelines and regulations. The ethical approval for all animal experiments was carried out in accordance with relevant guidelines and regulations by the ethical committee of the Friedrich Alexander University of Erlangen-Nuremberg (FAU), Germany.

### Mice

Mice were maintained at the specific pathogen-free animal care facility (FPZ) of the University of Erlangen-Nuremberg and housed in a room at 23 ± 2 °C, with 50 ± 10% humidity and a 12-h light/dark cycle (lights on from 08:00 a.m. to 08:00 p.m.). All mice were allowed free access to water and regular rodent chow.

### Ovariectomy-inducd osteoporosis (OVX)

Female C57BL/6JRj wild-type mice were obtained from Janvier Labs and experiments were performed according to guidelines of laboratory animal care and use. All efforts were made to reduce the number of animals tested and their suffering. For ovariectomy, mice (12 weeks of age) were anaesthetized with a ketamine-xylazine (KX) solution and bilaterally ovariectomized, while ovaries of the sham group were left intact. The ovariectomized mice were allowed to recover for two weeks to ensure the development of osteoporosis. After 6 full weeks of treatment with vehicle, 2d or GSK 2837808A, all animals were killed via cervical dislocation under CO_2_ anaesthesia and one tibia was excised for histological analyses and one for microcomputer tomography (μCT) imaging. Successful ovariectomy was examined at the day of preparation by anatomical analysis of the ovaries.

### Treatment with 2dG

Mice were divided into four groups: sham operated mice (sham, n = 6), ovariectomized mice (OVX, n = 6), 2dG treated OVX mice (OVX + 2dG, n = 7) and 2dG treated sham operated mice (sham + 2dG, n = 5). Mice were treated by intra-peritoneal injections with 2-Desoxy-Glucose (500 mg/kg/d body weight, suspended in aqua injectabile) daily for 6 weeks. Control groups were treated with a vehicle (aqu. inj.).

### Treatment with GSK 2837808A

Mice were treated by oral gavage with LDH-A inhibitor GSK 2837808A (8 mg/kg/d body weight, suspended in vehicle of 3% DMSO and 1% methylcellulose; Tocris Cat. No. 5189) daily for 6 weeks. Control groups were treated with vehicle (3% DMSO).

### Microcomputer tomography imaging (µCT)

Bones were fixed in 4% paraformaldehyde overnight prior to the analyses. All µCT imaging was performed using the cone-beam Desktop Micro Computer Tomograph “µCT 40” by SCANCO Medical AG, Bruettisellen, Switzerland (http://www.scanco.ch/en/systems-solutions/specimen/microct40.html). The settings were optimized for calcified tissue visualization at 55 kVp with a current of 145 µA and 200 ms integration time for 500 projections/180°. For the segmentation of 3D-Volumes an isotropic voxel size of 8,4 µm and an evaluation script with adjusted greyscale thresholds of the operating system “Open VMS” by SCANCO Medical was used. For 3D-μCT analysis, measurement was conducted 1680 µm ca. 400 µm below the approximate middle of growth plate, left or right tibia metaphysis.

### Cell culture

Bone marrow derived monocytes (BMDMs) were isolated as previously described and differentiated into osteoclast (OCs)^8^. Briefly, hematopoietic bone marrow cells were purified from tibial bone with a 70-μm cell strainer, cultured overnight and stimulated with appropriate growth medium (MEM Alpha Medium 1 × GlutaMAX, GIBCO; #32571-028, containing 10% L929 conditioned medium, 10% FCS and 1% penicillin/streptomycin) at 37 °C with 5% CO_2_. OCPs were cultured in OC-growth medium (MEM Alpha Medium 1 × GlutaMAX, GIBCO; #32571-028, containing 10% L929 conditioned medium, 10 ng/ml RANKL, 10% FCS and 1% penicillin/streptomycin) for 24 h. Osteoclast differentiation was quantified by counting multinucleated (> 3 nuclei) TRAP + cells on day 4 of OC culture using the Acid Phosphatase, Leukocyte (TRAP) Kit (Sigma-Aldrich, # 387A). Osteoclast fusion events were quantified by counting the nuclei per osteoclast.

### Calculation of the fusion index

For analysis of the fusion efficiency, a fusion index calculated. The total number of cell fusion events in one syncytium (number of nuclei/osteoclast) were counted (= fusion index). Multinucleated osteoclasts were defined as > 3 nuclei/cell. The absolute number of cells with the same amount of nuclei were set in ratio with the total number of cells per setting and given in cumulative frequency in percent in order to define the fusion efficiency.

### Bone resorption analysis

For analysis of osteoclast resorption, osteoclast differentiation was conducted in Osteoplates (Corning Osteo Assay Surface 24-well Multiple Well Plates; #3987). Experiments were stopped by aspirating the medium and adding 10% bleach solution for 5 min at room temperature. Afterwards, bleach solution was aspirated and cells were washed twice with 150 μl of dH2O and air-dried overnight. Pictures were taken with Zeiss Axioskop 2 microskop (5 × enlargement) and converted into black and white with Adobe Photoshop. Osteoclast resorption activity was quantified by measurement of black (= resorption area) versus white (= bone) pixels with a Histogram.

### Bone powder medium

Bovine bone meal box bone powder (from “Hund und Sport Hungenberg”; #KNM3) was crushed into fine fragments with sterile backed mortar. Bone powder was stored in the freezer. Needed concentrations were measured and filled up with not heat inactivated FCS. Tubes were heat inactivated in a water bath for 1 h at 56 °C. One sample was separated on two bacterial dishes and plated under UV light overnight. Finally 5 ml of heat inactivated and sterilized FCS containing Bone Powder was filled up with 45 ml of growth medium (MEM Alpha Medium 1 × GlutaMAX, GIBCO; #32571-028, containing 10% L929 conditioned medium, 10% FCS and 1% penicillin/streptomycin) to obtain bone powder medium.

### RT-PCR analysis

Total RNA was isolated from cells using peqGOLD TRIFast (peqlab, Germany). 1 µg was used for the first-strand complementary DNA synthesis (Amersham Biosciences), which was then used for SYBR Green–based quantitative RT-PCR as described previously^12^. Triplicates were performed according to the manufacturer’s instructions. The following mouse RT-PCR primer sequences were used:

**Table:** 

Primer	Forward sequence	Reverse sequence
beta-actin	TGTCCACCTTCCAGCAGATGT	AGCTCAGTAACAGTCCGCCTAGA
HK2	TTTCACCTTCTCGTTCCCCT	GTCATTCACCACAGCCACAA
PDK4	CCTTCACACCTCACCACAT	AAAGAGGCGGTCAGTAATCC
GLUT1	TCAACACGGCCTTCACTG	CACGATGCTCAGATAGGACATC
LDHA	GGACAGTGCCTACGAGGTGAT	GGATGCACCCGCCTAAGG
LDHB	GGGAAAGTCTCTGGCTGATGAA	CTGTCACAGAGTAATCTTTATCGGC
DC-STAMP	AAAACCCTTGGGCTGTTCTT	AATCATGGACGACTCCTTGG
OSCAR	TCGCTGATACTCCAGCTGTC	ATCCCAGGAGTCACAACTGC
CATH.K	ATATGTGGGCCAGGATGAAAGTT	TCGTTCCCCACAGGAATCTCT

### Extracellular flux assay

Real time bioenergetic profile of OCPs were obtained by measuring oxygen consumption rate (OCR) and extracellular acidification rate (ECAR) using the XFe96 extracellular flux analyzer (Seahorse Bioscience, Agilent Technologies, North Billerica, MA). Briefly, OCPs were seeded at a density of 150.000 cells per well (96-well-plate) and left untreated or treated with RANKL (10 ng/ml) in growth medium with and without bone powder for 48 h. On the day of measurement, cells were washed two times with Seahorse XF Base Medium and.

growth medium was replaced by Seahorse XF Base Medium and supplemented with glucose (10 mM), sodium-pyruvate (200 mM) and glutamax (200 mM). Further, medium was warmed up to 37 °C and pH was subsequently adjusted to 7.4 ± 0.1. For Glyco Stress assay, no glucose and pyruvat was added to Seahorse XF Basismedium. Following incubation in an incubator without CO_2_ at 37 °C for 60 min, basal OCR and ECAR were recorded for 105 min. Mito Stress assay was performed by sequential addition of 20 µM oligomycin (inhibitor of ATP synthesis), 10 μM carbonyl cyanide 4-(trifluoromethoxy) phenylhydrazone (FCCP, uncoupling agent) and 10 μM rotenone/antimycin A (inhibitors of complex I and complex III of the respiratory chain, respectively). Glyco Stress assay was performed by sequential addition of 100 mM glucose, 20 µM oligomycin and 500 mM 2dG. Parameters such as ATP-linked OCR, maximal OCR, spare respiratory capacity (SRC) and glycolysis were evaluated using the Wave XFe Analyzer software Wave Desktop 2.6 (PC only) and Wave Controller 2.6 (https://www.agilent.com/).

### Glucose and lactate measurements

Glucose and lactate content were determined as mg/dl in cell culture supernatants using a SuperGLcompact (Hitado, Möhnesee, Germany) according to the manufacturers standard operating procedure.

### Measurement of glycolysis and Krebs cycle metabolites

Phosphorylated intermediates and carboxylates were extracted with perchloric acid from xx to xx mg samples of shock-frozen xxx tissue for four biological replicates as described previously^13^ applying ionchromatography with an ICS3000 HPLC- system (Dionex) and ESI/MS/MS detection using a QTrap3200 Triple-Quadrupole massspectrometer with turbo V ion source (Applied Biosystems) operated in multiple reaction monitoring mode.

### Cytotoxicity assay

Cytotoxicity assay was performed with CytoTox 96 Non-Radioactive Cytotoxicity Assay from Promega as a measurement of lactate dehydrogenase (LDH) release into the supernatant according to the manufactors protocol. During experimental treatment via bone resorption assay, supernatant samples were cleared from non adherent cells and transferred to a 96 plate and an equal volume of CytoTox 96 reagent is added to each well and incubated for 30 min. Stop solution is added and the absorbance signal is measured at 490 nm in a plate reader. Results were calculated by subtracting the average values of the culture medium background from all values of experimental wells to compute percent cytotoxicity.

### Statistical analysis

Statistical analyses were performed using GraphPad Prism 8 software (https://www.graphpad.com/scientific-software/prism/). Results are depicted as median ± interquartile range (IQR) if not stated otherwise. For a two-group comparison, a Student's t-test was applied if the pretest for normality (D'Agostino-Pearson normality test) was not rejected at the 0.05 significance level; otherwise, a Mann–Whitney U-test for nonparametric data was used. *P* values less than 0.05 were considered significant. Results are expressed as mean ± SE (SEM). No statistical method was used to predetermine sample size.

## Supplementary information

Supplementary Figure 1.

Supplementary Legend.
