# Supplementary material for: Metabolic reprogramming of osteoclasts represents a therapeutic target during the treatment of osteoporosis
Source: Sci Rep. 2020 Dec 3;10:21020. doi: 10.1038/s41598-020-77892-4 (PMC7713370; doi:10.1038/s41598-020-77892-4)

Metabolic reprogramming of osteoclasts represents a therapeutic target during the treatment of osteoporosis

[1,2]Jule Taubmann, [1,2]Brenda Krishnacoumar, [1,2]Christina Böhm, [1,2]Maria Faas, [1,2]Dorothea I. H. Müller, [1,2]Susanne Adam, [1,2]Cornelia Stoll, [3]Martin Böttcher, [2,3]Dimitrios Mougiakakos, [4]Uwe Sonnewald, [4]Jörg Hofmann, [1,2]Georg Schett, [1,2]Gerhard Krönke* and [1,2]Carina Scholtysek*

*both authors equally contributed to this work

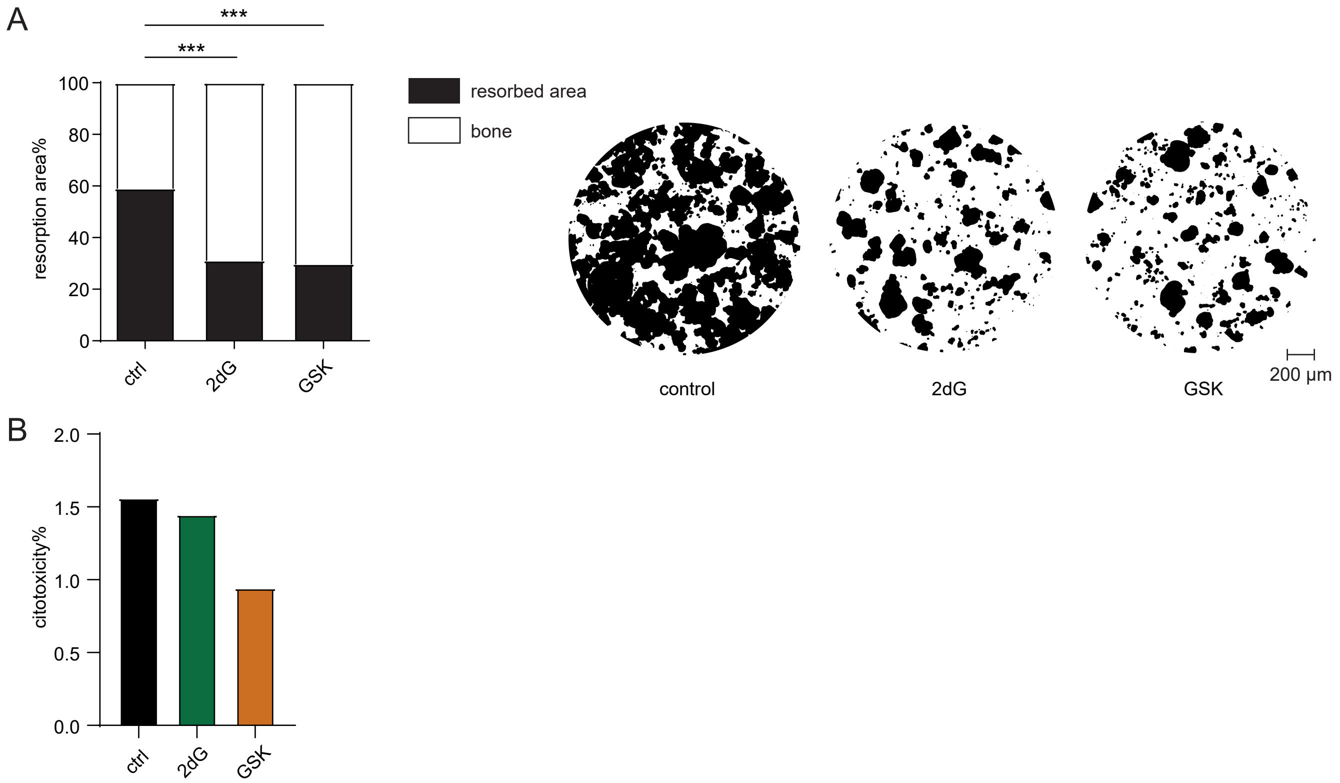

Supplement: Supplementary file 1 — Supplementary Figure 1. [file 41598_2020_77892_MOESM1_ESM.pdf]
