# Supplementary material for: Metabolic reprogramming of osteoclasts represents a therapeutic target during the treatment of osteoporosis
Source: Sci Rep. 2020 Dec 3;10:21020. doi: 10.1038/s41598-020-77892-4 (PMC7713370; doi:10.1038/s41598-020-77892-4)
Supplement: Supplementary file 2 — Supplementary Legend. [file 41598_2020_77892_MOESM2_ESM.docx]

**Supplementary Figure1: Inhibition of bone resorbing activity by GSK in vitro**

(A) OCs were generated on bone resorption plates for 5 days and stimulated with 2dG (10µM) or GSK (1µM). The percentage of the resorbed area was quantified via Photoshop and pictures show representative images of resorption pit formation. (B) Measurement of cytotoxicity in the supernatants after stimulation of OCs with RANKL (ctrl) and 2dG (10µM) or GSK (1µM).
